# Telomere shortening in late‐life depression: A potential marker of depression severity

**DOI:** 10.1002/brb3.2255

**Published:** 2021-06-21

**Authors:** Ana Paula Mendes‐Silva, Erica Leandro Marciano Vieira, Gabriela Xavier, Lucelia Scarabeli Silva Barroso, Laiss Bertola, Efrem Augusto Ribeiro Martins, Elisa Macedo Brietzke, Sintia Iole Nogueira Belangero, Breno Satler Diniz

**Affiliations:** ^1^ Centre for Addiction and Mental Health (CAMH) Toronto Ontario Canada; ^2^ Department of Morphology and Genetics Federal University of São Paulo São Paulo São Paulo Brazil; ^3^ LINC‐Interdisciplinary Laboratory of Clinical Neurosciences Federal University of São Paulo São Paulo São Paulo Brazil; ^4^ Graduate Program in Molecular Medicine Federal University of Minas Gerais School of Medicine Belo Horizonte Minas Gerais Brazil; ^5^ Department of Psychiatry Queen's University School of Medicine Kingston Ontario Canada; ^6^ Centre for Neuroscience Studies (CNS) Queen's University Kingston Ontario Canada; ^7^ Department of Psychiatry Faculty of Medicine University of Toronto Toronto Ontario Canada; ^8^ UConn Center on Aging University of Connecticut Health Center Farmington Connecticut USA; ^9^ Department of Psychiatry University of Connecticut Health Center Farmington Connecticut USA

**Keywords:** aging, cellular senescence, late‐life depression, telomere length

## Abstract

**Objectives:**

Telomeres are structures at the extremity of chromosomes that prevents genomic instability, and its shortening seems to be a hallmark of cellular aging. Past studies have shown contradictory results of telomere length (TL) in major depression, and are a few studies in late‐life depression (LLD). This explores the association between TL as a molecular marker of aging and diagnosis of LLD, the severity of depressive symptoms, and cognitive performance in older adults.

**Methods/design:**

We included 78 older adults (45 with LLD and 33 nondepressed controls, according to DSM‐V criteria), aged 60–90 years. TL was measured in leukocytes by a quantitative polymerase chain reaction, determining the relative ratio (T/S) between the telomere region copy number (T) and a single copy gene (S), using a relative standard curve.

**Results:**

TL was significantly shorter in the LLD compared with control participants (*p* = .039). Comparing groups through the severity of depressive symptoms, we found a negative correlation with the severity of depressive symptoms (Hamilton Depression Rating Scale‐21, *r* = −0.325, *p* = .004) and medical burden (*r* = −0.271, *p* = .038). There was no significant correlation between TL and cognitive performance (Mattis Dementia Rating Scale, *r* = 0.152, *p* = .21).

**Conclusions:**

We found that older adults with LLD have shorter telomere than healthy controls, especially those with a more severe depressive episode. Our findings suggest that shorter TL can be a marker of the severity of depressive episodes in older adults and indicate that these individuals may be at higher risk of age‐associated adverse outcomes linked to depression.

## Graphical abstract

Our study explores and examines the relationship between telomere length (TL) and Late‐Life Depression (LLD), considering the severity of depressive episodes. We found that TL was significantly shorter in the LLD than control participants and was negatively correlated with the severity of depressive symptoms and medical burden. Our findings suggest that shorter TL can be a marker of the severity of depressive episodes in older adults and indicate that these individuals may be at higher risk of age‐associated adverse outcomes linked to depression.

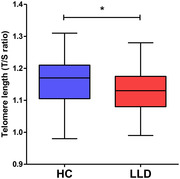

## 1 INTRODUCTION

Major depressive disorder (MDD) is common in older adults (aka late‐life depression [LLD]) with a prevalence of around 4% (Byers et al., 2010). It is a complex, heterogeneous disorder, influenced by both individual susceptibility (e.g., genetic predisposition) (Sullivan et al., 2000) and environmental factors (e.g., the experience of traumatic and stressful life events) (Byers et al., 2010; Kendler et al., 1995). Individuals with LLD usually experience cognitive impairment, more significant functional disability, more medical comorbidity burden, and have a higher risk for frailty compared with young adults with MDD (Diniz, 2018; Sibille, 2013; Verhoeven et al., 2014; Wolkowitz et al., 2011; Wolkowitz et al., 2011). The increase in aging‐related somatic conditions has been hypothesized to be a consequence of accelerated biological aging in the depressed population (Wolkowitz et al., 2011; Wolkowitz et al., 2010). There are different hypothesis for the emergence of a prematurely aged phenotype in LLD, including the dysregulation of glucocorticoid cascade, increased allostatic load, and telomere shortening (Blackburn et al., 2015; Diniz, 2018; Diniz et al., 2017; Diniz et al., 2018; Squassina et al., 2019).

Telomeres are structures of nucleoprotein complexes at the ends of linear chromosomes that consist of a variable number of tandem repeats of a double‐stranded TTAGGG nucleotide sequence and a 3′‐rich single‐stranded overhang (Lu et al., 2013). The telomere structure prevents end‐to‐end recombination, degradation, and fusion, having a critical role in maintaining the chromosomal integrity (Lu et al., 2013). Due to the inability of the DNA polymerase complex to replicate the 3′‐end of the lagging strand linear chromosomes, telomere length (TL) gradually decreases with every cell division (Tian et al., 2019). When the TL reaches a critical length, cell functioning becomes unstable, leading to senescence and a higher risk of cell apoptosis (Collado et al., 2007; Manoliu et al., 2018; Willeit et al., 2010). Telomere shortening is associated with age in most somatic tissues (Aubert, 2014) and is influenced by genetic and epigenetic regulation, cellular stress, and inflammation (Ridout et al., 2016). TL shortening has been linked to numerous age‐related diseases such as type 2 diabetes mellitus, cardiovascular disease, neurodegenerative diseases, and premature aging syndromes (Blackburn et al., 2015; Goglin et al., 2016; Herrmann et al., 2018; Zhao et al., 2013).

Previous studies investigated the association between TL and MDD in young adults, with most studies showing a reduction in TL among depressed individuals (Darrow et al., 2016; Lin et al., 2016; Ridout et al., 2016; Squassina et al., 2020; Vance et al., 2018; Wolkowitz et al., 2011). However, few studies focused on the older adult population. In a study including older adults with clinically significant depressive symptoms and nondepressed controls, TL was not significantly different between groups (Rius‐Ottenheim et al., 2012; Schaakxs et al., 2015). TL was also not associated with the depressive episode (e.g., symptoms severity, number of depressive episodes, or the age of onset of the first episode). However, there is robust evidence suggesting that LLD is associated with enhanced molecular senescence changes and dysregulation in biological pathways implicated in aging (e.g., dysregulation of proteostasis, proinflammatory activation, metabolic control) (Diniz, 2018; McKinney et al., 2012).

The main objective of this study was to investigate the association between TL and LLD. We also evaluated the association between TL with specific characteristics of the depressive episode (e.g., symptoms severity, cognitive impairment, and medical comorbidity burden). We hypothesized that participants with LLD would have shorter telomeres and TL would be associate with worse cognitive performance, more severe depressive symptoms, and higher medical comorbidity burden.

## 2 MATERIALS AND METHODS

### 2.1 Sample recruitment and assessments

We included a convenience sample of 78 older adults (45 with LLD and 33 healthy controls [HC]), aged 60–90 years, in this study. All patients with LLD were recruited and evaluated at the Psychogeriatric Outpatient Clinic at the Federal University of Minas Gerais, Belo Horizonte, Brazil, after referral to assess depressive symptoms. Typical aging older adults without a history of depression were included as a control group. They were recruited as part of an ongoing cohort study of healthy cognitive aging at the Federal University of Minas Gerais. All participants were above 60 years old at the time of study recruitment. All participants underwent a comprehensive psychiatric, clinical, and neurological assessments.

The psychiatric assessment included the administration of the Mini Neuropsychiatric Interview (Sheehan et al., 1998). The diagnosis of LLD was based on the DSM‐5 (Diagnostic and Statistical Manual of Mental Disorders–Fifth Edition) criteria for a major depressive episode (single and recurrent). The severity of depressive symptoms were rated with the Hamilton Depression Rating Scale‐21 items (HDRS‐21) (Hamilton, 1960). Inclusion criteria for controls were no history of MDD or other major psychiatric disorders, absence of sensory deficits, and no evidence of cognitive impairment based on the Dementia Rating Scale (DRS) scores. No participants (LLD and controls) were under current antidepressant treatment at the time of psychiatric assessment and blood collection. Additional exclusion criteria for this study were the presence of unstable medical illness, history of autoimmune disease, chronic use of anti‐inflammatory drugs medication, and history of substance abuse disorder in the past year.

We administered the Mattis DRS for neurocognitive assessment and excluded potential dementia cases in this population (Marson et al., 1997). The DRS provides a total score for cognitive performance and has been widely used in studies with older adults with different neuropsychiatric disorders. The sociodemographic and clinical characteristics of the samples are shown in Table 1.

**TABLE 1:** Characteristics of late‐life depressed and normal control study participants

	**HC (*n* = 33)**	**LLD (*n* = 45)**	**Test value**	***p***
**Demographics**				
Age, mean (SD)	70.8 (7.8)	72.5 (7.1)	*t* = 1.053	.300
Female sex, *n* (%)	33 (100)	41 (93.2)	*X*^2^ = 15.41	**<.0001**
Years of education, mean (SD)	8.8 (5.6)	5.1 (3.5)	*t* = 2.479	**.018**
**HDRS‐21, mean (SD)**	1.1 (1.3)	19.4 (6.4)	*t* = 18.73	**<.0001**
**CIRS‐G, mean (SD)**	4.7 (2.3)	8.4 (4.9)	*t* = 3.745	**.002**
**Mattis Dementia Rating Scale, mean (SD)**	134.4 (7.7)	120.7 (16.2)	*t* = 3.095	**.005**
**Telomere length (T/S ratio)**	1.2 (0.1)	1.1 (0.1)	*t* = 2.1	**.039**

HDRS‐21: Hamilton Depression Rating Scale‐21 items; CIRS‐G: Cumulative Illness Rating Scale – Geriatrics.

^a^ Numerical variables analyzed using independent samples *t*‐tests and categorical variables analyzed using *X* ^2^ statistics.

This study was approved by the local ethics committee at the Federal University of Minas Gerais, Belo Horizonte, MG, Brazil. Subjects signed written informed consent before their inclusion in the study.

### 2.2 Blood collection and DNA extraction

Peripheral blood was collected by venipuncture in EDTA tubes, and DNA was extracted from blood samples (45 LLD and 33 HC) following high salt method (Lahiri & Schnabel, 1993). DNA was quantified using a NanoDrop 1000 Spectrophotometer (NanoDrop Technologies, Waltham, MA/USA), then immediately stored at−80°C.

### 2.3 Telomere length

The DNA was diluted to 50 ng/μl. Leukocyte TL was measured by quantitative polymerase chain reaction (q‐PCR), previously described by Cawthon (2009). This technique consists in determining the relative ratio (T/S) between the telomere region copy number (T) and a single copy gene (S), using a relative standard curve. The ratio is proportional to the mean leukocyte TL in the peripheral blood of the participant. All reactions were made in triplicate, using Viia7 Real‐Time PCR System (Thermo Fisher Scientific). We used a single‐copy gene amplicon primer set for single‐copy gene albumin (5′–3′) (CGGCGGCGGGCGGCGCGGGCTGGGCGGAAATGCTGCACAGAATCCTTG and GCCCGGCCCGCCGCGCCCGTCCCGCCGGAAAAGCATGGTCGCCTGTT) and a telomere‐specific amplicon primer set (5′–3′) (ACACTAAGGTTTGGGTTTGGGTTTGGGTTTGGGTTAGTGT and TGTTAGGTATCCCTATCCCTATCCCTATCCCTATCCCTAACA).

Telomere and control gene assays yielded similar amplification efficiencies. It should be noted that the same control DNA sample from a healthy young individual was used as a control to generate a dilution curve on every plate. The intraassay variation was assessed by comparing the relative telomere estimates (T/S ratio) across assays for the positive controls, which were assayed on every assay plate and was never higher than 10%. The interassay maximum rate accepted was 15%.

### 2.4 Statistical analysis

Demographic and clinical variables distribution did not follow a normal distribution and were log‐transformed before the inferential statistical analyses. Chi‐square or *t*‐tests were done to test for differences between groups for categorical and continuous variables, respectively. We used a *t*‐test to evaluate for differences in TL between LLD and HC. Finally, the association between TL, demographic, and clinical variables was assessed by Pearson correlation tests. A *p* value <0.05 was considered to be significant. All analysis were conducted using SPSS v.25 (SPSS, Inc., Chicago, IL, USA).

## 3 RESULTS

The demographic and clinical characteristics of the sample are summarized in Table 1. LLD participants had significantly fewer years of education, higher medical comorbidity burden, and worse cognitive performance than the control group. Women were overrepresented in the LLD group, and there were no men included in the control group.

**FIGURE 1:**
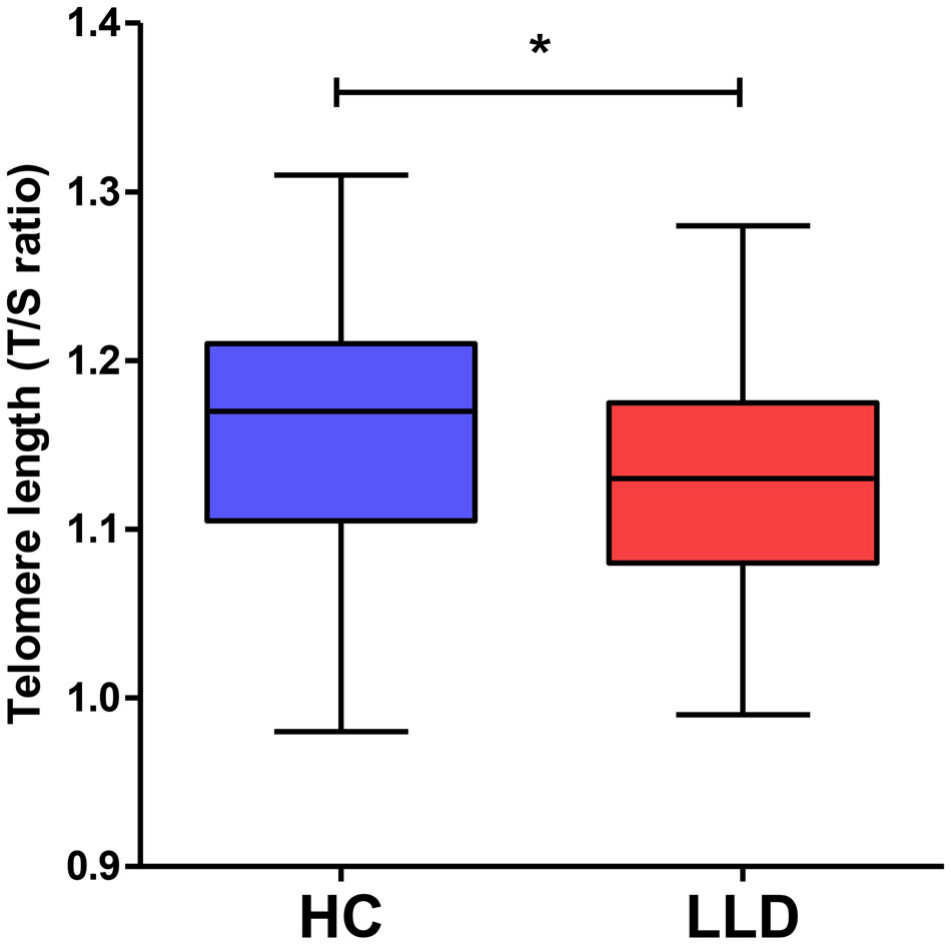
Individuals with LLD have shorter telomere lengths than age‐matched control subjects. A box plot of the mean telomere length (T/S ratio) score for both groups is displayed. The *y*‐axis represents telomere length in (T/S ratio) score scale. The *x*‐axis represents the health control (HC) and late‐life depression (LLD) groups

LLD participants had significantly shorter TL than controls (*p* = .039) (Table 1 and Figure 1). As there were no men included in the control group, we carried out a sensitivity analysis by excluding men participants in the LLD group and the results remained unchanged. TL was negatively correlated with the severity of depressive symptoms (*r* = −0.325, *p* = .004) (Figure S1), and medical comorbidity burden (*r* = −0.271, *p* = .04). There was no significant correlation between TL and age (*r* = 0.056, *p* = .6), years of education (*r* = 0.17, *p* = .13), and cognitive performance (DRS total score, *r* = 0.159, *p* = .21). However, after controlling for gender, years of education, cognitive performance, and medical comorbidity, the difference in TL was not statistically significant between groups (*F*(1,44) = 2.20, *p* = .145).

**FIGURE 2:**
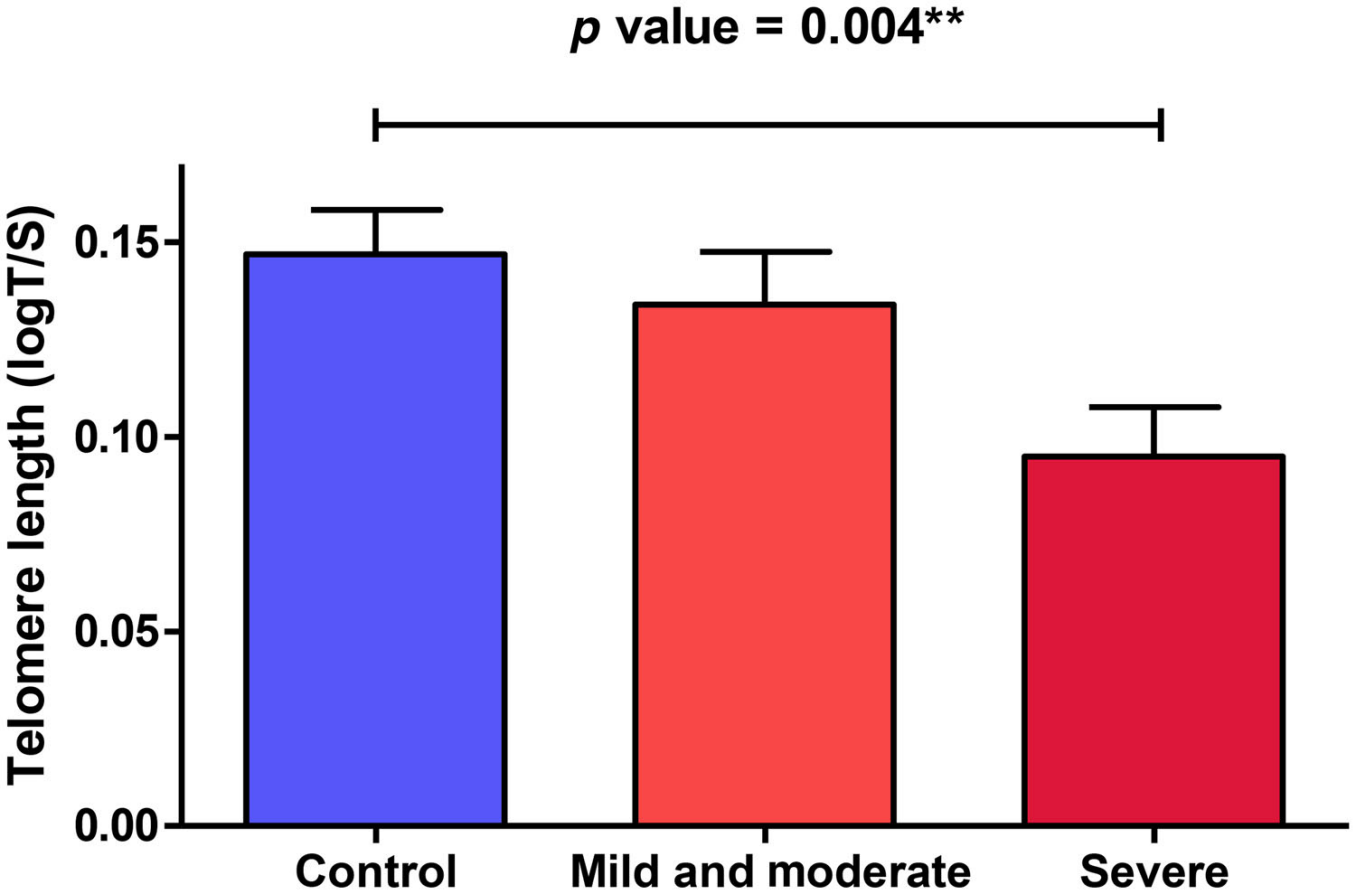
Mean telomere length (logT/S) for control subjects and LLD cases separated by depression severity (Hamilton Depression Rating Scale 21 items (HDRS‐21) scores). LLD—Severe depression group was significantly different from the control group (*p* value = .004)

As the correlation between the severity of depressive symptoms with TL was statistically significant, we divided the LLD participants according to the severity of the depressive episode based on the HDRS‐21 scores (mild, 8–16; moderate, 17−23; severe, ≥24). Those with severe depressive episodes presented shorter TL than controls, after controlling for potential confounding variables (*p* = .004) (Figure 2).

## 4 DISCUSSION

In this study, we investigated the association between TL and LLD. Our main findings were the significant negative correlation between TL and depressive symptoms severity, and that those with severe depressive episode showed a significantly shorter telomere compared to those with mild/moderate symptoms and controls. Our results are in line with previous studies in the literature focusing on younger adults with major depression (Lin et al., 2016; Ridout et al., 2016; Vance et al., 2018); however, it contrasts with previous reports in the elderly population (Rius‐Ottenheim et al., 2012; Schaakxs et al., 2015). Possible explanations for the divergent results include methodological differences between studies, like study setting, ascertainment of depressive symptoms, and sample characteristics (e.g., medical comorbidity, socioeconomic differences). On the other hand, these contrasting results also shed light on the potential heterogeneity of the biological changes related to depressive disorders in the elderly.

Our results extend recent evidence suggesting that LLD is associated with more intense age‐related biological changes (Brown et al., 2018; Diniz et al., 2017). Telomere shortening has been associated with adverse health outcomes across the lifespan, including more significant medical comorbidity and higher mortality risk (Aubert, 2014; Vakonaki et al., 2018; Vance et al., 2018; Wang et al., 2018; Wolkowitz et al., 2010). Our results showed a significant association between TL, depressive symptoms severity, and medical burden consistent with the previous findings (Darrow et al., 2016; Ghimire et al., 2019; Lindqvist et al., 2015; Price et al., 2013), and corroborates the hypothesis that telomere shortening can be a potential mechanism linking LLD an increased risk for mortality in this population (Diniz et al., 2014; Wolkowitz et al., 2011).

Increased psychological and chronic stress can culminate in cellular aging by DNA damage (Lindqvist et al., 2015; Wolkowitz et al., 2010). Substantial evidence supports the association of depressive disorder with abnormalities in stress‐related biological systems, such as the hypothalamic–pituitary–adrenal (HPA) axis (Tomiyama et al., 2012) and inflammatory responses (Glaser & Kiecolt‐Glaser, 2005). Moreover, depression is related to oxidative stress (Diniz et al., 2018) that can reduce telomerase activity and decrease neurotrophic factors (Manoliu et al., 2018), increasing apoptosis, senescence response, and reducing stem cell proliferation (Zhang et al., 2016). The persistence of unresolved senescence stimuli and their recurrence over time can lead to the accumulation of senescent cells, and at the biological level, it can manifest as a lower capacity to recover from stressor (Diniz, 2018; Diniz et al., 2017) accelerating the brain aging in LLD (Mendes‐Silva et al., 2019). In older persons, the accumulation of lifetime exposure to those unbalanced stress response systems could provide a basis for cellular exhaustion, leading to a senescence‐associated secretory phenotype, culminating in telomere shortening and accelerated aging observed in our study.

Emerging evidence supports TL as a biomarker of stress‐related cellular aging as a secondary mechanism to increased oxidative damage (Lindqvist et al., 2015; Wolkowitz et al., 2011). Several studies have shown that TL is reduced in patients with depression, including late‐onset, compared with healthy older adults. Vance et al. (2018) showed in a 2 years longitudinal study that MDD was associated with shorter leukocyte TL (LTL) (*b* = −0.55 ± 0.24, *F* (1, 115) = 5.14, *p* = .03), and no significant associations between LTL and symptom severity or duration were found. Pisanu et al. (2019) showed in their results a significantly reduced LTL in MDD subjects (*b* = −0.22, *p* = .02) when compared with controls, and the difference found was not influenced by any other clinical variable explored. Two recent meta‐analysis (Ridout et al., 2016; Schutte & Malouff, 2015) reported a significant correlation between depression and TL, and Squassina et al. (2020) study found shorter TL in MDD patients compared with controls (*p* value = .039) as well as increased inflammatory load. However, other studies also reported no association between TL and depression, including the absence of an association with the severity of the depressive symptoms (Rius‐Ottenheim et al., 2012; Schaakxs et al., 2015). The hypothesis of the association between TL and depression seems to depend on the severity of the depressive symptoms (Needham et al., 2015; Ridout et al., 2016), length of exposure to the depressive disorder (Hartmann et al., 2010; Needham et al., 2015), and other characteristics of the depressive episode commonly observed in LLD, like higher medical comorbidity burden or cognitive impairment (Diniz et al., 2014; Diniz et al., 2015; Wolkowitz et al., 2011).

The current results should be viewed in light of the study limitations. First, we included a relatively small sample size of LLD and controls, especially after splitting the LLD group according to the severity of the disease. The cross‐sectional design limit the generalization of our findings, and we can not draw any causal link between telomere shortening and LLD. We also did not measure the telomerase activity that is associated with TL maintenance. Therefore, we could not address if there is also dysregulation of the biological processes accountable for the maintenance of telomere biology in LLD. It should be noted that age was not significantly correlated with TL in our sample. Although most studies show a significant correlation between chronological age and TL, there are also reports of a nonsignificant association between these variables (Mather et al., 2010). One possible explanation is the limited age range of our cohort. Finally, we did not have a detailed neuropsychological assessment, and we could not explore the association between TL and cognitive impairment in LLD. The inclusion of medication‐free patients is a strength of our study and reinforce our results.

In conclusion, we showed that LLD is associated with telomere shortening, especially in those with a severe depressive episode. This result is in line with the literature and suggests that individuals with more severe depressive episodes present more pronounced biological aging than mild ones (Diniz et al., 2019). Future studies, with a longitudinal design, should be done to evaluate the prognostic significance of TL shortening in the LLD, especially related to treatment response, multimorbidity and mortality risk, and incidence of dementia.

## CONFLICT OF INTEREST

All authors declared no conflicts of interest.

### PEER REVIEW

The peer review history for this article is available at https://publons.com/publon/10.1002/brb3.2255.

## Supporting information

SUPPORTING INFORMATION

SUPPORTING INFORMATION

## Data Availability

Data are available on request from the authors.
