# Supplementary material for: Telomere shortening in late‐life depression: A potential marker of depression severity
Source: Brain Behav. 2021 Jun 21;11(8):e2255. doi: 10.1002/brb3.2255 (PMC8413729; doi:10.1002/brb3.2255)

Supplementary figure 2 - Pearson correlation between medical burden and telomere length. CIRS-G: Cumulative Illness Rating Scale – Geriatrics

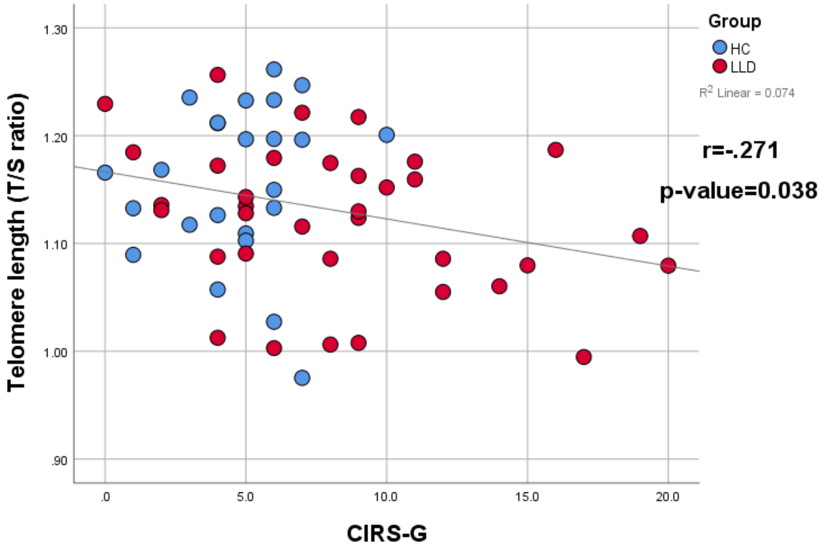

Supplement: Supplementary file 2 — SUPPORTING INFORMATION [file BRB3-11-e2255-s002.pdf]
